# Automatic identification of triple negative breast cancer in ultrasonography using a deep convolutional neural network

**DOI:** 10.1038/s41598-021-00018-x

**Published:** 2021-10-14

**Authors:** Heng Ye, Jing Hang, Meimei Zhang, Xiaowei Chen, Xinhua Ye, Jie Chen, Weixin Zhang, Di Xu, Dong Zhang

**Affiliations:** 1 The MOE Key Laboratory of Modern Acoustics, Department of Physics, Nanjing University, Nanjing, 210093 China; 2 Department of Ultrasound, The First Affiliated Hospital of Nanjing Medical University, Nanjing, 210029 China; 3 The State Key Laboratory of Acoustics, Chinese Academy of Science, Beijing, 10080 China

**Keywords:** Cancer, Biomarkers, Medical research, Engineering

## Abstract

Triple negative (TN) breast cancer is a subtype of breast cancer which is difficult for early detection and the prognosis is poor. In this paper, 910 benign and 934 malignant (110 TN and 824 NTN) B-mode breast ultrasound images were collected. A Resnet50 deep convolutional neural network was fine-tuned. The results showed that the averaged area under the receiver operating characteristic curve (AUC) of discriminating malignant from benign ones were 0.9789 (benign vs. TN), 0.9689 (benign vs. NTN). To discriminate TN from NTN breast cancer, the AUC was 0.9000, the accuracy was 88.89%, the sensitivity was 87.5%, and the specificity was 90.00%. It showed that the computer-aided system based on DCNN is expected to be a promising noninvasive clinical tool for ultrasound diagnosis of TN breast cancer.

## Introduction

Breast cancer is one of the main causes of cancer deaths in women^1^. Most breast cancers begin in the ducts, some begin in the lobules, while a small number start in the other tissues^2^. Early diagnosis of breast cancer is urgent for improving the prognosis of patients and prolonging their survival^3^. The B-mode ultrasonic image is an important clinical method to observe the internal structures of biological tissue due to its non-ionizing, noninvasive, and low-cost nature. With Breast Imaging Reporting and Data System (BI-RADS) by the American College of Radiology, ultrasound provides the information about the structure and characteristics of masses, including shape, echo pattern, orientation, margin, etc.^4^. However, the training and years of experience that can affect the diagnostic performance of the radiologists^5,6^.

According to gene expression profile of estrogen receptor (ER), progesterone receptor (PR), and/or human epidermal growth factor receptor 2 (HER2), breast cancer can be divided into four molecular subtypes (luminal-A, luminal-B, HER2 overexpression and basal-like)^7–9^. Triple negative (TN) breast cancer is a distinctive type of breast cancer characterized by the absence of ER, PR, and HER2 receptor expression^10–12^. Although pathological biopsy has been widely used to identify molecular subtypes of breast cancer, it has the limitation of underestimation and overestimation owing to the spatial and temporal heterogeneity of the tumor^13^. It has reported that ultrasound imaging could capture the biological and molecular characteristics of the tumor^14,15^. TN breast cancer is a genetically diverse, highly heterogeneous, and rapidly evolving disease, which is associated with a relatively young age, invasive histological and clinical behavior with poor prognosis outcome^12,16,17^. Several retrospective studies related to conventional ultrasound image characteristics of TN breast cancer showed that compared with non-triple negative (NTN) breast cancer, TN breast cancer was more likely to show benign features, such as oval or round shape, smooth or circumscribed margin, and was less likely to have an echogenic halo^18–20^. In this sense, there may be false-negative results in TN breast cancer evaluation, which might lead to delayed diagnosis and a potentially worse clinical outcome^20^. As a non-invasive method, artificial intelligence (AI) provides comprehensive anatomical information of the tumor, and objectively describes the relationship between ultrasound image and biological characteristics of breast cancer^21,22^. In view of the fact that TN breast cancer is more sensitive than other subtypes to preoperative chemotherapy, AI based identification of TN breast cancer is of great importance in clinical diagnosis and treatment.

Previously, in order to perform TN breast cancer evaluation, some studies based on magnetic resonance (MR) images were reported. Agner et al.^23^ used linear discriminant analysis combined with support vector machine on dynamic contrast material–enhanced MR images, and found good discrimination of TN cancers from NTN cancers. In some ultrasound image related studies, machine learning had been used to evaluate TN breast cancers. Wu et al.^24^ analyzed 140 breast masses ultrasound images (including 23 cases of TN breast cancer and 117 cases of NTN breast cancer), and concluded that machine learning can distinguish ultrasound images between TN and NTN breast cancers. Compared with traditional machine learning methods with limited accuracy, deep convolution neural network (DCNN)^25–28^ with the advantage of automatic feature extraction and accurate classification has been widely used in medical field in recent years, i.e. ophthalmology^29^, dermatology^30^, orthopedics^31^, etc.^32–34^, which has shown similar performance to doctors. In order to test whether the application of DCNN can improve the accuracy of computer aided diagnosis of breast masses, 1844 breast mass greyscale ultrasound images were retrospectively analyzed in this work and a deep learning-based algorithm was developed for automated detection of benign or malignant breast mass. Furthermore, the ability of DCNN to distinguish TN breast cancers from NTN breast cancer was also discussed.

## Results

### Patients

**Figure 1:**
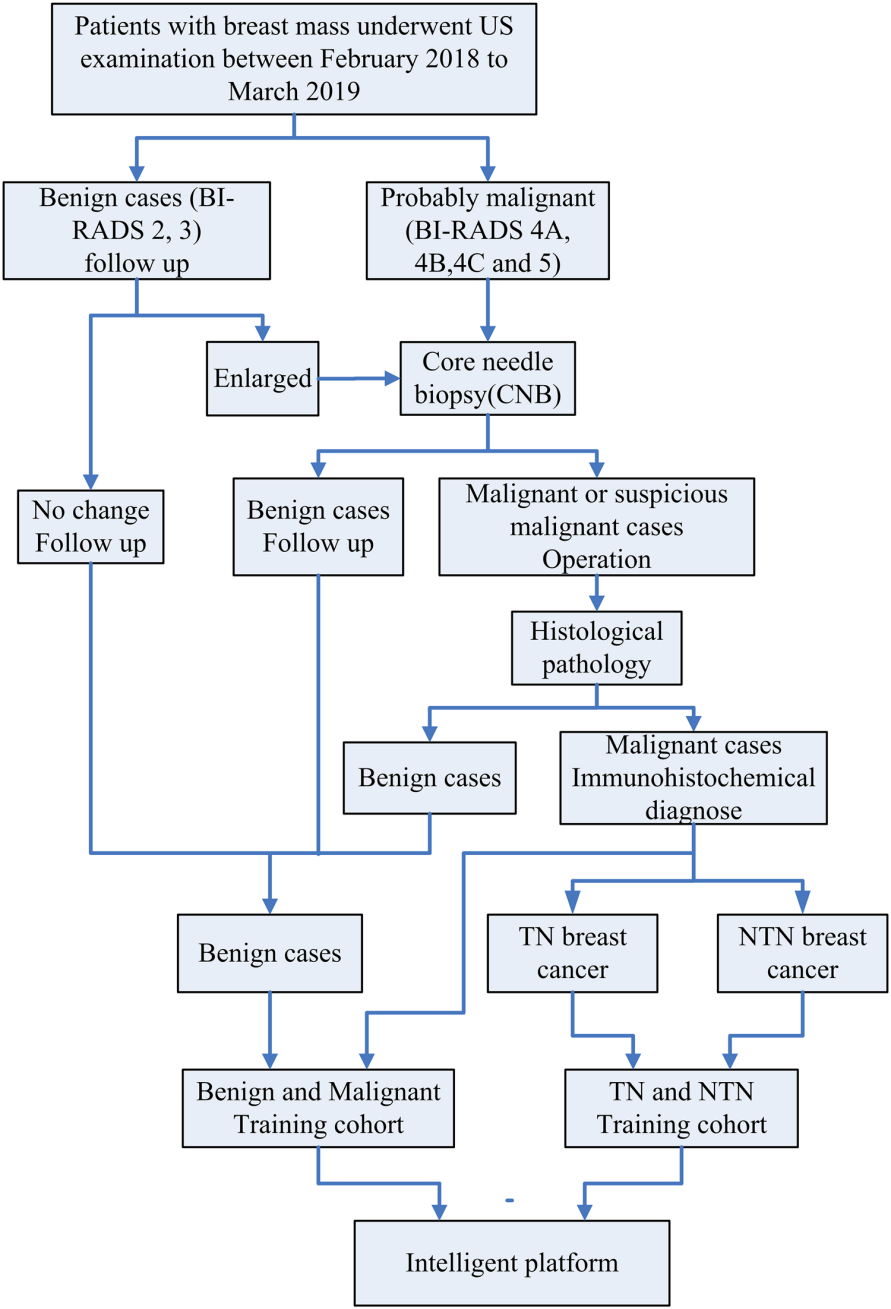
Inclusion criteria for the study cohorts and experiment procedure.

This retrospective study reviewed the patients underwent breast ultrasound examination between February 2018 and March 2019 in the First Affiliated Hospital of Nanjing Medical University, China. Ultrasound data were also obtained from Chinese medicine Hospital of Jiangsu Province as external test. The study was approved by the institutional review committee of the First Affiliated Hospital of Nanjing Medical University and Chinese medicine Hospital of Jiangsu Province. The informed consent was obtained from all patients. All research methods were conducted in accordance with the ethical guidelines of the Helsinki Declaration. In the present study, the B mode ultrasound images were used. The criteria of inclusion were as follows: (a) older than eighteen years old; (b) patients underwent biopsy or operation with determinate histopathologically or immunohistochemically; (c) no preoperative treatment or intervention (radiotherapy, chemotherapy, ablation) before ultrasound examination. In total, 1618 images of 1261 patients comprised the benign and malignant training cohort and a test cohort of 226 images from 185 patients was screened with the same criteria from the First Affiliated Hospital of Nanjing Medical University. Patients were grouped into training and test cohorts randomly. All the data, including age, sex, pathologic findings, and ultrasound reports, were derived from the Picture Archiving and Communication Systems (PACS) system. For the TN and NTN training cohort, we randomly selected 102 images of TN breast cancers diagnosed by immunohistochemistry, and randomly selected 102 images of NTN breast cancers at the same time. We randomly selected 8 images of TN breast cancers and 10 images of NTN breast cancers to form the test set. External test cohort includes 29 TN and 28 NTN images were from Chinese medicine Hospital of Jiangsu Province. Figure 1 shows the inclusion criteria for the cohort in this study and the experiment procedure.

### Examination technique

The enrolled ultrasound images were mainly performed with three commercially available ultrasound machines: (1) Esaote (Genova, Italy; twice MyLab). (2) Siemens (Buffalo, USA; S3000). (3) Philips (Amsterdam, Netherlands; EPIQ 7), and a small numbers data were from other manufacturers. A linear array probe with a frequency bandwidth of 6–15 MHz was used for this study. In the ultrasound examination, the patient was placed supine, exposing the bilateral breasts, and then scanned laterally, longitudinally, and obliquely. All the US examinations were performed by three radiologists (J.H., J.C., and W.X.Z) with fifteen, ten and six years of experience in breast ultrasound respectively.

### Image analysis

Two radiologists (J.H., J.C.) performed independent interpretations blinded to pathology results. Interpretations were performed by the evaluation of the primary breast cancer ultrasound images according to the BI-RADS with typical characteristics, including the mass size, shape, orientation, margin, echo pattern, the presence of calcifications. If the classification of the two radiologists was the same, it would be regarded as the final result of the radiologist's interpretation. If the two radiologists (J.H., J.C.)’ result did not agree with each other, extra interpretation needed to be performed by the third radiologist (X.H.Y) with twenty five years of experience in breast ultrasound.

The histological and immunohistochemical results were used as gold standard in this study. The core needle biopsy (CNB) was performed on solid breast masses categorized as probably malignant (BI-RADS classes 4A, 4B, 4C and 5). A 14G core needle was used under ultrasound guidance. After the operation, radiotherapy or chemotherapy were performed on malignant breast mass. Follow-up was performed on breast masses with BI-RADS classes 2 and 3, which was recommended in clinical routine^4^. And the breast masses which remained unchanged for more than 2-year follow-up were taken as benign images in the study.

### Demographics and histopathologic findings

**Figure 2:**
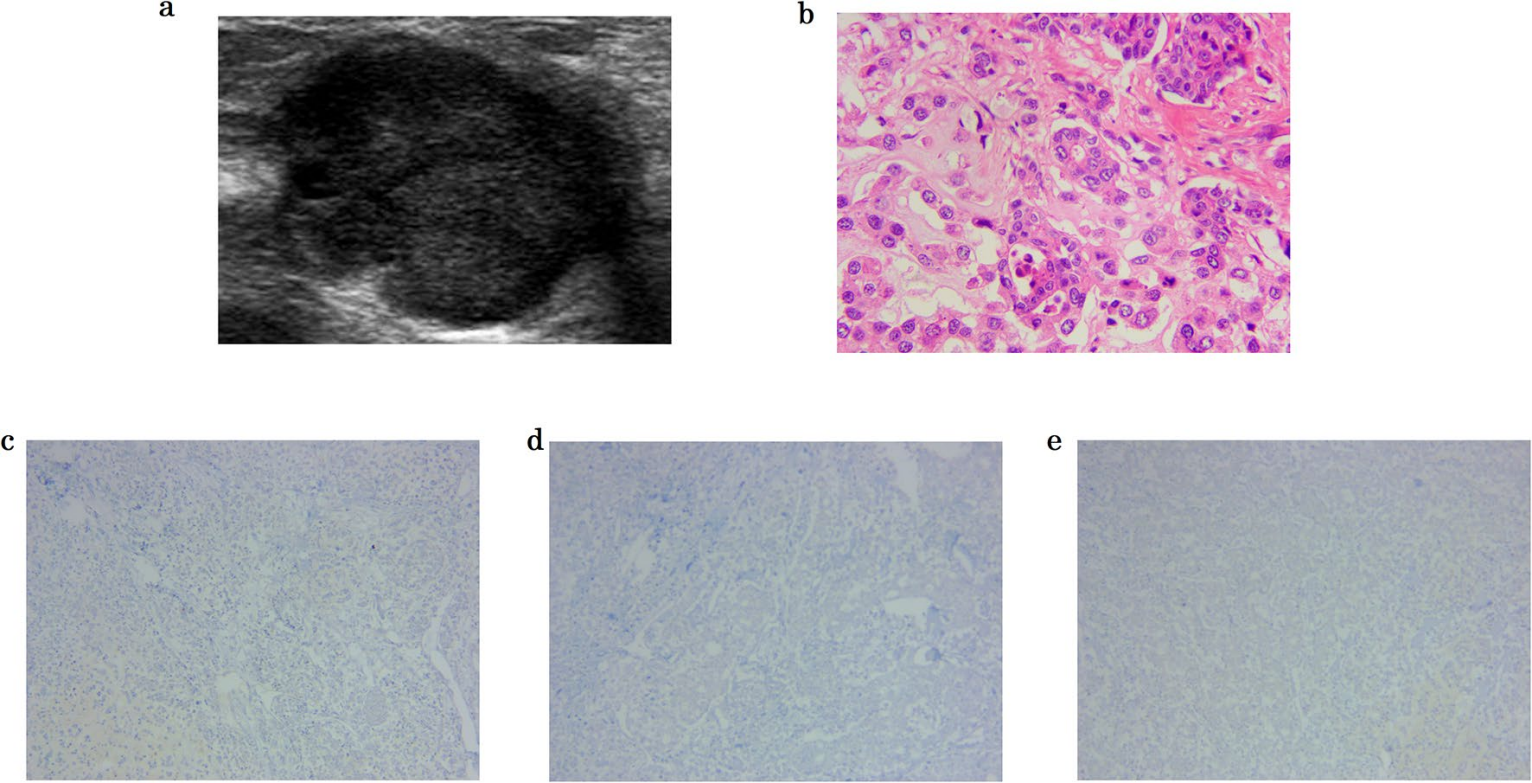
Images in a 52-year-old woman with triple-negative breast cancer (TNBC). (**a**) Grey-scale US images presenting with an irregular shape mass suspicious for cancer. (**b**-**e**) Pathological images revealed invasive ductal cancer (**b**: HE*400, **c**: absence of estrogen receptor, **d**: absence of progesterone receptor, **e**: absence of human epidermal growth factor receptor 2, G: Ki-67 70%).

Note. mean data are mean ± standard deviation.

In this study cohort, a total number of 1844 images extracted from 1446 patients were enrolled in this retrospective study. All the pathological results were summarized in Table 1. The first training data consisted of 1618 images from 1261 patients, including 820 images for benign cohort (598 female, mean age 39.65 ± 10.36 ys) and 798 images for malignant cohort (3 male, mean age 48.30 ± 6.70 ys and 660 female, mean age 52.80 ± 11.50 ys). The 226 images in the first test set were acquired from 185 patients, including 90 benign images (79 female, mean age 40.54 ± 10.23 ys) and 136 malignant images (106 female, mean age 51.02 ± 11.97 ys). The malignant images in the first test set included 40 TN images and 96 NTN images. All the basic information of selected images were collected in Table 2. Figure 2 shows images for a 52-year-old woman with TN breast cancer, where Fig. 2a was grey-scale US images presenting with an irregular shape mass suspicious for cancer; Fig. 2b was pathological images with HE*400; Fig. 2c,d and 2e showed the absence of estrogen receptor, progesterone receptor, and human epidermal growth factor receptor 2.

**Table 1:** Pathological types of collected cases.

Benign		Malignant	
BIRADS II, III	800	Invasive ductal carcinoma	805
Breast fibroadenoma	33	Intraductal carcinoma	65
Intraductal papilloma	6	Mucinous breast cancer	15
Fibrocystic breast disease	8	Breast carcinoma in situ	16
Breast adenosis	13	Invasive lobular carcinoma	19
Mastitis	4	Breast papillary carcinoma	9
Breast cyst	40	Sarcomatoid carcinoma	1
Cyclomastopathy	5	Metaplastic breast arcinoma	4
Benign lobulated tumor I	1

**Table 2:** The basic information of collected cases.

All masses	Training masses	Test masses
Benign	820 images	90 images
	598 females , 39.65 ± 10.36 ys	79 females, 40.54 ± 10.23 ys
Malignant	798 images (70 TN, 728 NTN)	136 images (40TN, 96 NTN)
	3 males, 48.30 ± 6.70 ys	106 females, 51.02 ± 11.97 ys
	660 females, 52.80 ± 11.50 ys

### Benign and malignant

In this study, an artificial neural network were developed, in which the boundaries of the masses were not required to be drawn by the radiologist. The mass packing by surrounding the calipers used in the clinic for mass measurement was used. A DCNN was trained and used to classify benign and malignant breast masses, and the results were compared with those reported by the radiologist based on BI-RADS.

First, a nine fold cross validation was used to verify the validity and performance of this algorithm. Table 2 lists basic information of the images. Those imaging data were used to train the network and determine the hyper parameter of the network. The training data were divided into nine sub-datasets, and ninefold validation were performed. The averaged area under the receiver operating characteristic curve (AUC) of the algorithm was 0.9504 (95% CI 0.9321, 0.9687). The averaged accuracy was 91.02% (95% CI 89.49%, 92.55%), the averaged sensitivity was 90.23% (95% CI 87.89, 92.56), and the averaged specificity was 91.76% (95% CI 89.91%, 93.61%). (Table 3).

**Table 3:** The result of cross validation (benign vs. malignant).

Round	Accuracy (%)	Sensitivity (%)	Specificity (%)
1	90.23%	91.76%	88.76%
2	91.47%	93.40%	89.52%
3	92.27%	89.47%	95.35%
4	91.40%	90.32%	92.47%
5	93.98%	92.21%	95.51%
6	91.85%	92.22%	91.49%
7	92.00%	92.13%	91.86%
8	87.65%	85.54%	89.66%
9	88.30%	85.00%	91.21%
Average	91.02% (95% CI 89.49%, 92.55%)	90.23% (95% CI 87.89%, 92.56%)	91.76% (95% CI 89.91%, 93.61%)

**Figure 3:**
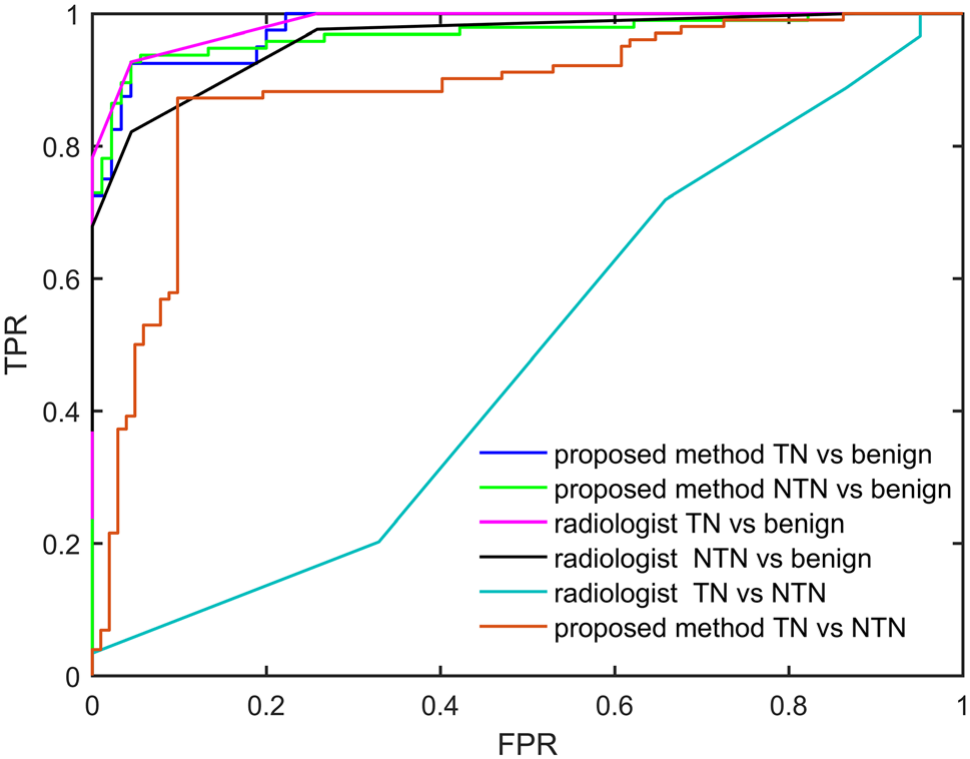
AUC of the proposed algorithm. TPR (True Positive Rate), FPR (False Positive Rate).

Then, differentiating benign and malignant masses for the test set (226 images from 185 patients) was performed using the trained algorithm. There were 90 benign images, 40 TN breast cancer images and 96 NTN breast cancer images. The results showed that the AUC of discriminating TN breast cancer from benign ones was 0.9789, the accuracy was 94.62%, the sensitivity was 92.50%, and the specificity was 95.56%. The AUC of discriminating NTN breast cancer from benign ones of the proposed method was 0.9689, the accuracy was 93.01%, the sensitivity was 90.62%, and the specificity was 95.56% (Fig. 3).

For comparison, the test set were also evaluated by the radiologist according to BI-RADS. The AUC of radiologist’s discriminating TN breast cancer from benign ones was 0.9857, the accuracy was 94.44%, the sensitivity was 92.73%, and the specificity was 95.51%. The AUC of radiologist’s discriminating NTN breast cancer from benign ones of the proposed method was 0.9598, the accuracy was 89.02%, the sensitivity was 82.14%, and the specificity was 95.51%. Statistical analysis showed that there was no significant difference between the algorithm and that of expert radiologist (*p* > 0.5)^35,36^.

### TN and NTN

A 18 fold cross validation was used to verify the validity and performance of this algorithm. 204 images (102 NTN breast cancer, and 102 TN breast cancer) were used to train the network and determine the hyper parameter of the network. The training data were divided into 18 sub-datasets, and cross validation was performed. The AUC of the algorithm was 0.8746 (95% CI 0.8347, 0.9145) (Fig. 3). The averaged accuracy was 88.75% (95% CI 86.31%, 91.19%), the averaged sensitivity was 87.35% (95% CI 83.27%, 91.44%), and the averaged specificity was 90.32% (95% CI 85.83%, 94.80%). The AUC of radiologist’s discriminating triple negative breast cancer from non-triple ones was 0.4461, the accuracy was 53.80%, the sensitivity was 71.91%, and the specificity was 34.15% (Table 4).

**Table 4:** The result of cross validation (TN vs. NTN).

Round	Accuracy (%)	Sensitivity (%)	Specificity (%)
1	90.91	100.00	83.33
2	83.33	83.33	83.33
3	91.67	83.33	100.00
4	83.33	83.33	83.33
5	84.62	83.33	85.71
6	81.82	83.33	80.00
7	91.67	100.00	83.33
8	90.91	83.33	100.00
9	81.82	83.33	80.00
10	90.00	80	100
11	92.86	85.71	100
12	90.90	100	83.33
13	100	100	100
14	90.91	80	100
15	90	80	100
16	90.00	100	80
17	90.91	83.33	100
18	81.82	80	83.33
Average	88.75% (95% CI 86.31%, 91.19%)	87.35% (95% CI 83.27%, 91.44%)	90.32% (95% CI 85.83%, 94.80%)

10 NTN breast cancers, and 8 TN cancers were tested. The AUC of discriminating TN breast cancer from NTN ones was 0.9000, the accuracy was 88.89%, the sensitivity was 87.50%, and the specificity was 90.00%. 29 TN samples and 28 NTN samples obtained from Chinese medicine Hospital of Jiangsu Province were used for the external test, the AUC is 0.8817, the accuracy is 89.94%, the sensitivity is 86.67%, and the specificity is 93.33%.

## Discussion

### Benign and malignant

In recent years, due to an increased need for efficient and objective evaluation of ultrasound images, artificial intelligence ultrasound diagnosis has been widely studied. The computer-aided system based on deep learning, due to its self-learning ability, has achieved good results in diagnosing breast masses. Fujioka et al.^37^ retrospectively analyzed 480 benign and 467 malignant breast mass ultrasound images, and constructed deep learning model of differentiating breast masses by convolution neutral network architecture GoogLeNet, and achieved an AUC of 0.913. Byra et al.^38^ introduced ImageNet-pretrained VGG19 with fine-tuning and matching layer at input based on a set of 882 breast mass ultrasound images to classify breast mass, and reached an AUC of 0.936. Though these studies achieved high accuracy and proved to be a useful tool for breast mass classification, fewer images were used, especially for malignant masses. In this study, a larger cohort including 1844 images were retrospectively analyzed to develop an artificial neural network, and the AUC of the algorithm reached 0.9504. The result of the algorithm was consistent with those reported by previous studies, and demonstrated that deep learning technology could help radiologists to classify breast masses in ultrasound images.

### TN and NTN

While molecular markers can be evaluated from tissues obtained in biopsy or surgery, it is invasive and subject to the tissue sampling bias problem. TN breast cancer is associated with aggressive histology, poor clinical prognosis, unresponsiveness to usual endocrine therapies and shorter survival. If it is possible to predict the presence of TN breast cancer based on noninvasive ultrasound features, this information will be beneficial for both pretreatment planning and prognosis, and will add to the understanding of the biological behavior of this disease. Nowadays, much attention focusing on deep learning methods has been paid to TN breast cancer using MR images. Agner et al.^24^ used linear discriminant analysis combined with support vector machine classifier, achieved an AUC of 0.73 (95% CI 0.59, 0.87) for TN breast cancer versus NTN breast cancer. Koo et al.^39^ found the usefulness of computer-aided breast MR diagnosis in predicting the level of tumor-infiltrating lymphocytes in TN breast cancers. However, there were few studies focused on intelligent recognition of breast cancer ultrasonic images at the genetic and cellular levels. Guo et al.^40^ reported an automatic radiomics approach to assess the associations between quantitative ultrasound features and biological characteristics. They used a support vector machine classifier with three-fold-cross-validation to evaluate a strong correlationship between ultrasound features and biological characteristics. Wu et al.^24^ discriminated 140 cases with logistic regression including 23 TN and 117 NTN, and the specificity was 82.05%, the sensitivity was 78.26%. However, the enrolled images were extracted from a manually drawn region of interest in those studies. In comparison, the boundaries of the masses were not required to be drawn in this work, which could overcome the defect of operator dependence and avoid to add additional work to radiologist. The results of our study showed that the AUC of discriminating TN breast cancer from NTN ones was 0.9000, the accuracy was 88.89%, the sensitivity was 87.50%, and the specificity was 90.00%, which showed an improvement of accuracy.

### The low accuracy of radiologists distinguishing between TN vs NTN

The AUC of radiologist’s discriminating TN breast cancer from non-triple ones was 0.4461, the accuracy, sensitivity and specificity were also very low. Because we used the BI-RADS score of the radiologists during the usual B-mode ultrasound diagnostic routine process, and it did not differentiate molecular subtypes. This was the reason for the low diagnosis efficiency of radiologists in distinguishing between TN vs NTN.

### Originality & clinical significance

Restnet50 was proposed in Ref 24, in this paper we did some fine-tuned work to adapt to our specific task.

The accuracy of discriminating benign and malignant breast mass ultrasound images was a little higher than the previous work. One reason is that we collected more training data. The other reason is the power of deeper layers of Resnet and our fine-tuned techniques help to achieve more characteristic of the B-mode breast mass ultrasound images.

The main clinical significance of this paper is discriminating TN breast cancer from non-triple ones. To predict the presence of TN breast cancer with B-mode ultrasound features, it will be beneficial for pretreatment planning and prognosis. Because it is noninvasive, it can reduce the suffering of patients. And it will add to the understanding of the biological behavior of this disease.

### Bias of Selection of data

There is no bias of selection of data in the paper.

- Step 1: The 820 benign and 798 malignant ultrasound images for training, 90 benign and 136 malignant images for testing were randomly selected from the records between February 2018 and March 2019 in hospital. Further, we did ninefold cross validation, which showed the robustness of the algorithm.
- Step 2:102 for training and 8 for testing TN ultrasound images were the total patients in the hospital between February 2018 and March 2019. 102 for training and 10 for testing NTN ultrasound images were randomly selected from the malignant images used in Step 1. Then we enrolled 8 images of TN breast cancers and randomly selected 10 images of NTN breast cancers to form the test set. Patients were grouped into training and test cohorts randomly. Further, we did 18 fold cross validation, which showed the robustness of the algorithm.

There are different types of ultrasound machines in different hospitals, even in one hospital. To make sure the proposed method could be applied in different equipment, the original data from the different ultrasound machines were used. Tables 5 and 6 list the data distribution collected from different machines. It suggests that the proposed method could be applied to different ultrasound machines.

**Table 5:** Data distribution of ultrasound machines in discrimination of benign from malignant.

	Train, malignant	Train, benign	Test, malignant		Test, benign
			TN	NTN	
**Esaote**	534	651	27	76	41
**Philips**	33	163		2	45
**Simens**	230	1	11	17	/
**GE**	1	5	2	1	4
**Total**	798	820	136		90

**Table 6:** Data distribution of ultrasound machines in discrimination of TN from NTN.

	TN, train	NTN, train	TN, test	NTN, test
**Esaote**	77	81	6	9
**Philips**	5	5	/	/
**Simens**	11	16	2	1
**GE**	5	/	/	/
**Vinno**	1	/	/	/
SuperSonic	1	/	/	/
**Sumsung**	2	/	/	/
**Total**	102	102	8	10

### Limitations

This study still had some limitations. The sample size of this study was small and the data were derived from only a single center. The specificity of discriminating NTN from TN breast cancer using the proposed DCNN is around 90%, which means around 10% of tumors could be misdiagnosed. Future studies involving larger number of multi-center patients are warranted to demonstrate the viability of the proposed approach in clinical settings. If add more patients for verification, the accuracy of the model could be further verified and improved.

### Conclusion

In this paper, a deep learning-based algorithm was developed for the automated diagnosis of benign and malignant breast masses, and for further identification of TN breast cancer. The results showed the computer-aided system based on DCNN is expected to be a promising noninvasive clinical tool for ultrasound diagnosis of TN breast cancer.

## Materials and methods

### Pathologic and immunohistochemical examinations

All the enrolled patients undergoing pathological and immunohistochemical examinations were performed in the First Affiliated Hospital of Nanjing Medical University. The following biomarkers were routinely determined as immunohistochemical factors: ER, PR, HER2, Ki-67, epidermal growth factor receptor (EGFR), and cytokeratin (CK) 5/6. ER, PR, HER2, Ki-67 and EGFR immunohistochemical staining was performed on an automated Ventana BenchmarkXT slide stainer (Ventana, Tucson, AZ, USA), using primary antibodies against ER (prediluted, SP1, Ventana), PR (prediluted, 1E2, Ventana), HER2 (prediluted, 4B5, Ventana), Ki-67 (prediluted, MIB-1, Ventana), and EGFR (prediluted, 3C6, Ventana). CK5/6 (1:200, D5/16 B4, Dako, Carpinteria, CA, USA) immunohistochemical staining was performed on a Dako Omnis device (Dako). A cut-off value of ≥ 1% was used to define ER and PR positivity. The intensity of HER2 expression was scored semiquantitatively as 0, 1, 2, or 3. HER2-negative was classified with a score of 0 or 1.

### Fine-tuned DCNN

Convolutional neural network consists of a set of convolution and pooling operations applied to obtain complex features from the input image. These features are flattened into a vector. The output of the model is a collection of continuous variables that represented the predicted probabilities for each category. Deeper neural networks are more difficult to train. Resnet^28^, residual learning framework,was presented to ease the training of networks that are substantially deeper than those used previously. Resnet explicitly reformulate the layers as learning residual functions with reference to the layer inputs, instead of learning unreferenced functions. Comprehensive empirical evidence showed that Resnet are easier to optimize, and can gain accuracy from considerably increased depth.

In this work, the Resnet50 DCNN is fine-tuned for the diagnosis of breast masses. The detailed mechanism is as below.

(i) The building block is defined as
  1 $$y = F\left( {x, \left\{ {W_{i} } \right\}} \right) + x$$
  where *x* and *y* are the input and output vectors of the layers considered. The function $F\left( {x, \left\{ {W_{i} } \right\}} \right)$ represents the residual mapping to be learned. The operation *F* + *x* is performed by a shortcut connection.
(ii) The image density resolution is normalized to 8 bit density and 224 × 224 size.
(iii) To apply the network in this study, the last fully connected layer is changed for a global average pool layer, followed by a full connection layer and Softmax.
(iv) The weights are optimized by the adaptive learning rate (adadelta) optimization algorithm as:
  2 $$\Delta x = \frac{\partial f}{{\partial x}}/\frac{{\partial^{2} f}}{{\partial x^{2} }}$$
  3 $$\Delta x = - \frac{{RMS\left[ {\left( {\Delta x} \right)} \right]_{t - 1} }}{{RMS\left[ g \right]_{t} }}g_{t}$$
  4 $$E\left[ {\Delta x^{2} } \right]_{t} = \rho E\left[ {\Delta x^{2} } \right]_{t - 1} + \left( {1 - \rho \Delta x_{t}^{2} } \right)$$

It is optimized in 12 mini-batch size. The parameters, i.e. the learning rate and the maximum epoch number are set at 0.0001 and 148 respectively. The loss function is identified as binary cross entropy.

**Figure 4:**
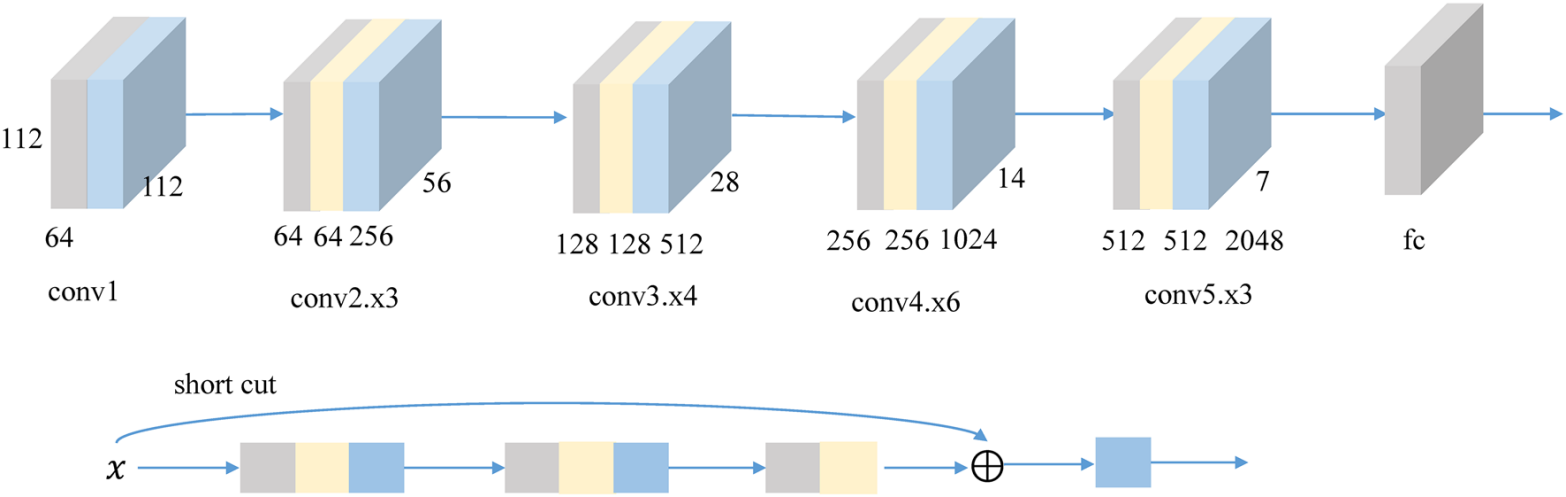
The network structure of the method.

Figure 4 shows the structure of the network. Tensorflow 2.0 and tensorboard are used to implement the DCNN. The training is conducted on computers equipped with Intel Core i5-8300h CPU and 8 GB memory, NVIDIA geforce GTX 1060 GPU and 6 GB memory.

### Statistical analysis

Matlab (Mathworks Corporation, USA) was used for data analysis. A T-test is performed on the assumption that the data in vector comes from a distribution with zero mean, and return the test result of H. H equals 0 indicates that the null hypothesis cannot be rejected at the significance level of 5%. H equals one means that the null hypothesis can be rejected at the level of 5%. Suppose the data comes from a normal distribution with unknown variance.

The confidence interval (CI) of the paired T-test: the CI of the mean of true population, which is returned as a two-element containing the upper and lower bounds of 100 × (1 -Alpha)% CI.
